# Efficacy of Auricular Acupressure for Chronic Low Back Pain: A Systematic Review and Meta-Analysis of Randomized Controlled Trials

**DOI:** 10.1155/2017/6383649

**Published:** 2017-07-18

**Authors:** Li-Hua Yang, Pei-Bei Duan, Qing-Mei Hou, Shi-Zheng Du, Jin-Fang Sun, Si-Juan Mei, Xiao-Qing Wang

**Affiliations:** ^1^Department of Oncology, Hospital Affiliated to Nanjing University of Traditional Chinese Medicine, 155 Hanzhong Road, Gulou District, Nanjing, Jiangsu 210029, China; ^2^Department of Nursing, Hospital Affiliated to Nanjing University of Traditional Chinese Medicine, 155 Hanzhong Road, Gulou District, Nanjing, Jiangsu 210029, China; ^3^School of Nursing, Nanjing University of Traditional Chinese Medicine, 138 Xianlin Road, Qixia District, Nanjing, Jiangsu 210046, China; ^4^Department of Epidemiology and Biostatistics, School of Public Health, Southeast University, 87 Dingjiaqiao Road, Gulou District, Nanjing, Jiangsu 210009, China

## Abstract

**Objectives:**

To identify the efficacy of auricular acupressure on pain and disability for chronic LBP by systematic review.

**Methods:**

A search of randomized controlled trials was conducted in four English medical electronic databases and three Chinese databases. Two reviewers independently retrieved related studies, assessed the methodological quality, and extracted data with a standardized data form. Meta-analyses were performed using all time-points meta-analysis.

**Results:**

A total of 7 trials met the inclusion criteria, of which 4 had the low risk of bias. The findings of this study showed that, for the immediate effect, auricular acupressure had large, significant effects in improving pain within 12 weeks. As for the follow-up effect, the pooled estimates also showed promising effect at 4-week follow-up after 4-week intervention (standardized mean difference = −1.13, 95% CI (−1.70, −0.56), *P* < 0.001). But, for the disability level, the therapeutic effect was not significant (mean difference = −1.99, 95% CI (−4.93, 0.95), *P* = 0.18). No serious adverse effects were recorded.

**Conclusions:**

The encouraging evidence of this study indicates that it is recommended to provide auricular acupressure to patients with chronic low back pain. However, a more accurate estimate of the effect will require further rigorously designed large-scale RCTs on chronic LBP for improving pain and disability.

## 1. Introduction

Low back pain (LBP) is now a prevalent and burdensome issue for both individuals and society, affecting approximately 60%–80% of the world's population, and 20% of them have developed into chronic symptoms [1, 2]. Eighty-five percent of patients in chronic LBP conditions have nonspecific underlying causes or pathology [3]. Besides persistent pain, declined physical activity is one of the major complaints in patients with chronic LBP. According to the Global Burden of Diseases, Injuries and Risk Factors Study 2010 (GBD 2010), “at the global level, among 291 diseases and injuries, LBP has been the main reason for disability and labelled as the highest cause of years lived with disability” [4]. In the United States, chronic LBP has resulted in huge negative effects on people's well-being and cost to society, as reflected by increased medical care costs and disability-related loss of productivity and wages [5, 6]. The continued high prevalence of LBP and serious socioeconomic burden relating to LBP may highlight the requirement for more effective, safe, and low-cost pain management. As one of these LBP managements, auriculotherapy (AT) may play an important role [7–10].

Auricular therapy, as defined by Oleson, refers to “a healthcare modality by stimulating the external surface of the ear to alleviate pathological conditions in other parts of the body” [11]. The French AT of Nogier and the Chinese are currently two main lines of research that can explain the principles of AT. Dr. Paul Nogier first determined the somatotopic arrangement of ear points as the inverted fetus in the 1950s and considered the auricular microsystem as reflexology of a neurological action [12, 13]. The Chinese school theorizes that using AT to treat disease should root in traditional Chinese medicine (TCM) [12, 14]. In TCM, the vital energy (*Qi*) of the body circulates in the channels and collaterals, and imbalance of a person's* Qi* may cause disease or illness. Stimulating a specific area of the auricular cartilage regulates* Qi*, activates the energy pathways, and has regulative effects on the corresponding zang-fu function [13, 14]. Means of stimulation on the auricular acupoints are either multitudinous, including small acupuncture needles [15],* Semen vaccariae* (a type of plant seed) [16], magnetic pellets [17], and electroacupuncture [18], or applied directly to the skin like transcutaneous electrical nerve stimulation (TENS) [19]. Unlike other models, the stimulation of* S. vaccariae* or magnetic pellets taped on the outer ear is conducted by pressing them with the thumb and forefinger. In this model, without any invasive procedure, auricular acupressure (AA) seems to be more accepted by patients, and also, related adverse events such as inflammation of the acupoints may have been reduced [20].

Recently, enthusiasm is growing for the role of AA in managing chronic LBP. Lots of clinical trials have been performed, and the results have shown promising effects [8, 21, 22]. Several systematic reviews (SR) of auricular therapy for pain management have been conducted [23, 24], mainly on calculating summary estimates from eligible trials at final time point (final time-point meta-analysis, FTM). In fact, for management of chronic pain, duration of treatment is a key factor for therapeutic outcomes [24]. Exploring all time-points meta-analysis (ATM) of repeated measures to capture the trend of effectiveness of AA over the time is inevitable and necessary. Up to now, two published systematic reviews have studied time effects [25, 26]. One of them is on the topic of traditional Chinese medicine treatments for neck pain and low back pain [25]. However, in the section of analyses consisting of acupressure for LBP, RCTs adopted auricular pressing therapy or acupressure in the whole human body as experimental strategies were all found for further analysis. The other one demonstrated the role of auriculotherapy in managing chronic pain, but this study involved varieties of implementation models (auricular acupuncture, auricular acupressure, and auricular electrostimulation), rather than using auricular pressing therapy only [26].

Given these conditions, we aimed to quantitatively assess the effects of AA for the management of chronic LBP from randomized controlled trials (RCTs) in terms of two major complaints: pain and disability as primary outcome(s). And, if possible, ATM would be used for meta-analysis.

## 2. Methods

### 2.1. Selection Strategy

With no time limit, four English databases (PubMed, Embase, the Cochrane Library, and AMED) and three Chinese databases (CBMdisc, CNKI, and WanFang Data) were searched until March 18, 2016, with the following Mesh terms and text words: (“auriculotherapy” OR “auricular therapy” OR “auricular point sticking” OR “auricular point therapy” OR “auricular plaster therapy” OR “auricular pressing therapy” OR “ear point” OR “auricular acupressure” OR “aural therapy” OR “ear acupressure”) AND (“low back pain” OR “backache” OR “lumbosacral”) AND (“randomized controlled trial” OR “random^*∗*^”). Finally, a snowball search was done, in which reference lists of eligible RCTs were screened and checked for potential relevant clinical studies. Non-English eligible publications were translated into English for further analysis and EndNote software was used to manage citations.

### 2.2. Selection of Studies

Only RCTs were selected as eligible studies. Further, they should satisfy the following criteria.

*P (Population)*. Studies that examined adults (≥18 years old) suffering from chronic nonspecific LBP which lasted for more than 3 months [27, 28] were reviewed. But those that recruited participants with any specific pathologies (i.e., with inflammatory, malignant disease or fracture) were ineligible.

*I (Intervention)*. Studies of interventions that adopted AA as experimental strategies or the primary modality in managing LBP were included. The taped objects can be botanical plant seeds (i.e.,* S. vaccariae*) or magnetic pellets. In particular, RCTs on the use of AA, which involve invasive techniques, such as auricular acupuncture, electroacupuncture stimulation, and* erjian* bloodletting method, were excluded.

*C (Comparison)*. The intervention to be compared with AA would include any of the following: conventional modalities, no treatment, sham, or other TCM.

*O (Outcome)*. Each eligible trial should take pain intensity (e.g., Visual Analogue Scale, Numerical Rating Scale) and disability measured by validated instruments (e.g., Roland-Morris Disability Questionnaire [29]) as its primary outcomes. Other outcomes, such as total therapeutic effect on chronic LBP (e.g., from the* Standards for Diagnosis and Curative Effect of Chinese Medical Symptom* or* Clinical Guideline of New Drugs for *TCM [30, 31]), would be considered as secondary indicators.

### 2.3. Study Outline

First, seven electronic databases were searched for relevant studies. After deleting the duplications, one reviewer (Li-Hua Yang) selected potential relevance based on title, abstract, or keywords and the other reviewer (Si-Juan Mei) read a random sampling independently. Then, both reviewers (Li-Hua Yang and Si-Juan Mei) reviewed the full texts of relevant studies. Finally, a snowball technique was utilized to check for more eligible RCTs from the reference lists of selected articles. During the processes above, disagreements between the two reviewers were resolved by using a consensus method.

### 2.4. Quality Critical Appraisal

Two reviewers (Li-Hua Yang and Si-Juan Mei) independently assessed the methodological quality using the Cochrane risk of bias tool for RCTs recommended by Cochrane handbook [32]. The Cochrane Collaboration's risk of bias tool consists of six items (randomization, allocation concealment, blinding, incomplete outcome data, selective outcome reporting, and other potential sources of bias). Each item was scored as “met,” “unmet,” or “unclear.” Because AA may not be double-blinded, in our review, we considered single-blinding of outcome assessors as “met” for blinding.

Based on Cochrane handbook, we divided the quality of RCTs into three levels (A, B, and C). “A” indicated that all or most of the six criteria were met, which stands for the low risk of bias. For one candidate RCT, if one or more criteria were partly met, B level would be rated representing unclear risk of bias. But if one or more criteria were not met, “C” reflecting the high risk of bias would be defined. Given that the high risk of bias would greatly reduce the credibility of the results, articles rated as C level would be eliminated.

Two reviewers (Li-Hua Yang and Si-Juan Mei) scored each RCT separately, with a third reviewer (PBD) acting as an arbiter when any disagreements occurred. If study contents were insufficient to determine the risk of bias, the corresponding author of the study would be contacted for further information.

### 2.5. Extraction of Data

Two reviewers (Li-Hua Yang and Si-Juan Mei) independently extracted the data using a standardized data form. Pieces of information about characteristics of the participants (e.g., sample size, age, sex, and specific conditions of participants), intervention protocol (e.g., taped objects, selected ear points, acupoint detection, detailed instructions of manual pressing, duration of AA, follow-up, and the control intervention), and therapeutic outcomes (including main outcome measure and the results, adverse events) were all recorded. Any disagreements between two reviewers were resolved by consensus and we tried to contact study authors of the RCTs to supply the missing data.

For continuous data on pain intensity, the changes from baseline of most eligible studies expressed with means and standard deviations (SD) could be extracted from the original or calculated according to methods recommended by the Cochrane handbook. So we would use the differences in change score for meta-analysis in order to make the results more credible.

### 2.6. Quantitative Synthesis of Data

Meta-analysis was accomplished using software RevMan (version 5.2). ATM of repeated measures would be applied to analyze the trend of effectiveness of AA on chronic LBP over time. And in our review, data were analyzed according to the duration of AA and follow-up, which were categorized as short-term (up to 4 weeks), long-term (12 weeks), or follow-up (about 4 weeks after intervention).

In each meta-analysis, the degree of heterogeneity among studies was estimated by using the *χ*^2^ statistics and *I*^2^ test with a *P* value < 0.10 and *I*^2^ > 50% of statistical significance. If the data was measured without significant heterogeneity (*I*^2^ < 50% and a *P* value > 0.10), a fixed-effect model would be used [33]. Otherwise, a random-effect model would be applied to pool the data if the trials are clinically homogenous enough, and then sensitivity analysis would be conducted to identify the sources for contributing heterogeneity. Or they would be synthesized with qualitative analysis rather than quantitative assessment. When a sufficient number of RCTs were available, a funnel plot would be constructed to examine potential publication bias.

Two summary statistics were used for meta-analysis of pain intensity, the mean difference (MD) and the standardized mean difference (SMD). If the pain scores were measured by the same scale, MD would be used in meta-analysis. But if they were measured by different scales, SMD would be applied. According to Cochrane handbook and Warsi et al. [32, 34], a magnitude effect size (SMD) of <0.2, 0.2~0.5, and >0.5 was, respectively, defined as small, moderate, and large effect. For dichotomous data, we calculated a relative risk (RR) and corresponding 95% Confidence Interval (CI).

## 3. Results

### 3.1. Search Process

**Figure 1:**
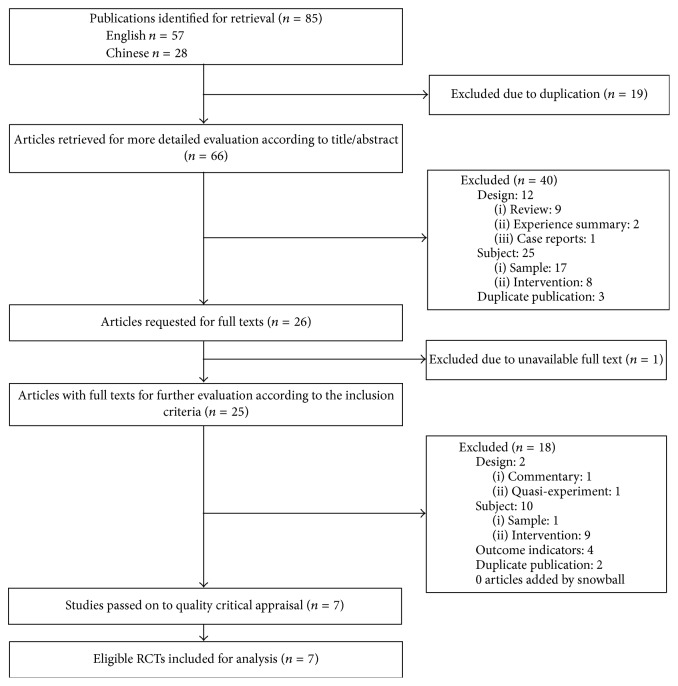
Study flow diagram.

We identified 85 potentially relevant records in total. After removing the duplicates, 66 records were retrieved for evaluation according to title/abstract. Excluded on title and abstract were 40 references, leaving 26 articles requested for full texts. With 1 study unavailable, 25 records with full texts were subsequently evaluated according to the criteria. At this stage, 18 records were excluded for various reasons presented in Figure 1. Meanwhile, no articles were selected based on snowball search. Therefore, in our review, 7 articles that met the inclusion criteria were passed on to quality critical appraisal [20, 35–40].

### 3.2. Critical Appraisal of Quality

Of 7 studies, we defined 4 RCTs as A quality level [20, 37–39] and 3 RCTs as B quality level according to the Cochrane risk of bias tool [35, 36, 40]. Disagreements between the two reviewers were solved after consensus meeting. The risk of bias assessment of each included trial is described in Table 1.

### 3.3. Characteristics of Eligible RCTs Included for Analysis

A total of 7 studies met the inclusion criteria and were passed on to analysis.  Table 2 contains the information of their characteristics. All RCTs were from USA (3) [37–39], Hong Kong Special Administrative Region of the People's Republic of China (1) [20], and mainland of China (3) [35, 36, 40], respectively. Six (6) were in English [20, 35–39], and one was in Chinese [40]. The dates of publication for the included 7 studies ranged from 2007 to 2015. Participants in four of the seven studies were middle-aged patients (2) or the elderly (2).

Studies varied in sample size. All eligible RCTs, with size ranging from 19 to 74, totally allocated 369 adult patients with chronic LBP or lumbar muscle strain. Of these RCTs, four studies compared AA with placebo control [20, 37–39] and another three used* Tai Chi* exercise [35]/conventional medicine [36]/walking training of lunge twist [40] as comparison.

### 3.4. Content and Implementation of AA

#### 3.4.1. Selection and Number of Ear Points in AA

Generally, auricular acupoints selected for treatment consist of two types: the main part and the adjunct. As presented in Table 3, the number of main ear points selected in all eligible studies ranged from 4 to 7. As for the adjunct part, only 2 RCTs claimed that adjunct ear points were selected according to syndrome differentiation [35, 40]. As a unique diagnostic method, differentiation of syndrome* (Bian Zheng)* in TCM aims to analyze and recognize the syndrome of disease for subsequent treatment decision. However, in the 2 RCTs referring to selection of adjunct ear points, there was no further information described on types of syndrome. Both of them reported that 2 to 3 adjunct acupoints were added as required. In four RCTs [20, 35, 36, 40], the AA treatment was given to only one ear each time, with two ears treated alternately. While in the other 3 studies, during each week, bilateral auricular points were treated for 5 days. And prior to the next treatment there are 2 days left without taping the ears so as to recover the acupoint's sensitivity and minimize the risk of an allergic reaction to the tape [37–39].

In all included studies, 15 main auricular acupoints were commonly used for treating chronic LBP.* Shenmen* (7/7) and subcortex (6/7) were the ear points of high-frequent use, which were considered primarily for alleviating pain, followed by lumbosacral region (5/7), liver (4/7), kidney (4/7), sympathetic (3/7), low back (2/7), waist (2/7), popliteal fossa (1/7), groove of spinal posterior (1/7), sciatic nerve (1/7), urinary bladder (1/7), buttock (1/7), spleen (1/7), and* Ashi point* (1/7), respectively. For the nomenclature and locations of auricular points, as is known, among the 93 auricular acupuncture points of the international standard of auricular acupuncture points (AAPs) developed by the World Federation of Acupuncture-Moxibustion Societies (WFAS), 34 (36.6%) were based on the nomenclature and locations of Nogier, twenty-one (22.6%) were based on the anatomical terminology of the surface of the auricle, and the remaining 38 (40.8%) were based on Chinese AAPs [41]. According to this standard, we reviewed all auricular acupoints used for treating chronic LBP in 7 included studies and found that 3 RCTs were simultaneously based on the nomenclature and locations of Nogier, the anatomical terminology of the surface of the auricle, and the Chinese auricular acupuncture points which were closely combined with clinical practice [37–39]. The remaining 4 were only based on the nomenclature system put forth by Nogier and the Chinese [20, 35, 36, 40].

#### 3.4.2. Use of Taped Objects and Acupoint Detection Tools in AA

Six of the eligible studies used* S. vaccariae* as taped objects in AA [35–40]. In addition to* S. vaccariae*, magnetic pellets were also commonly applied in China. In our review, one study used AA treatment in both experimental group and control group [20], but the participants in the intervention group were taped with magnetic pellets while those in control group were given* S. vaccariae*.

Exploring and locating the hypersensitive spots has been one of the key points in auricular plaster therapy. Four RCTs used an electrical acupoint finder working on the principle of detecting decreased-resistance points [20, 37–39]. Ear points could be identified when the electrical detector made a sound indicating the corresponding location on the body. Two RCTs applied a probe [35, 40] and the remaining one did not report the use of detection tools [36].

#### 3.4.3. Instructions of Manual Pressing

According to Li et al., as a taped object,* S. vaccariae* has been confirmed to have no therapeutic effect without exerting manual pressure [42]. Therefore, demonstration of manual pressing technique to participants is the most frequently used supplementary modality in AA therapy. 85.7% of the RCTs (6/7) adopted it while the remaining one reported that participants in control group* (S. vaccariae)* or experimental group (magnetic pellets) were all reminded not to press on it in order to avoid a confounding effect due to physical pressure [20].

The instructions on manual pressing mainly consisted of its frequency, duration, timing, and the intensity required. Four of the six RCTs with pressing implementations reported the frequency and duration of pressing completely [36–39]. Three of them claimed it should be done “not less than 3 times per day” and for “3 minutes per acupoint every time” [37–39]. The other one reported the frequency should be “3 times per day,” but the instruction on duration was described as “3–5 minutes per acupoint every time” instead of clarifying a specific length of time [36]. Two RCTs only stated the duration of pressing without daily frequency [35, 40]. Both of them emphasized that each selected acupoint should be lightly kneaded 20 times and kneading should be kept for 20 minutes per time. Regarding the timing for manual pressing, only three studies mentioned it should be done for 3 minutes whenever pain occurred in addition to routine frequency of pressure [37–39]. As for the intensity, 2 RCTs considered patient's subjective feelings of* “de qi”* which presented as soreness, numbness, distention, heaviness, or hotness and simultaneously stressed that the pressing force should be based on the patients' tolerance [35, 40]. One RCT declared that mild pressure was used for initial therapy and the following intensity should be tolerated by patients [36]. Other 3 RCTs considered moderate pressure as suitable intensity but the implementation of manual pressing was not described specifically [37–39].

#### 3.4.4. Duration and Follow-Up of AA

All eligible RCTs gave the explicit information of intervention duration. Of these studies, the duration of auricular plaster therapy ranged from 2 weeks to 3 months. However, 71.4% (5/7) had a duration of interventions not more than 4 weeks [20, 36–39], among which 4-week duration was the most common length (3/7, 42.9%) [37–39].

Among 7 RCTs, 4 articles did not have a plan for a follow-up visit [35, 36, 38, 40], and the follow-ups of the 3 RCTs are from 2 weeks to 1 month [20, 37, 39].

#### 3.4.5. Compliance and Attrition Rate

The therapeutic effect of AA can be greatly affected by the quality of manual pressing (i.e., the time and frequency of seed pressing). However, only three articles reported the compliance of participants to the instructions of manual pressing [37–39]. Four RCTs reported their attrition rates which are from 9.5% to 37.8% [36–39].

### 3.5. Outcome Analysis

#### 3.5.1. The Chronic Back Pain Intensity

**Figure 2:**
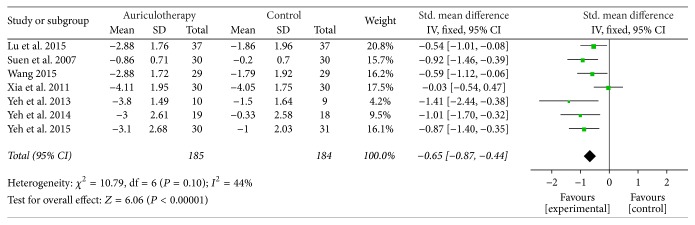
Forest plot of pain relief of AA at all final time points.

*The Immediate Effect after Intervention*. In 7 RCTs, the most frequently used scales for chronic LBP were Visual Analogue Scale (VAS), Verbal Rating Scale (VRS), and the Brief Pain Inventory short form (BPI-sf) (Table 4). Three of them were all measured on a scale of 0 to 10, with higher score indicating more severe pain intensity. Data of changes from baseline were available or calculated. The result of meta-analysis revealed that, compared with control group, AA group had a large, significant effect in relieving pain at all final time points [SMD = −0.65, 95% CI (−0.87, −0.44), *P* < 0.001] (Figure 2).

**Figure 3:**
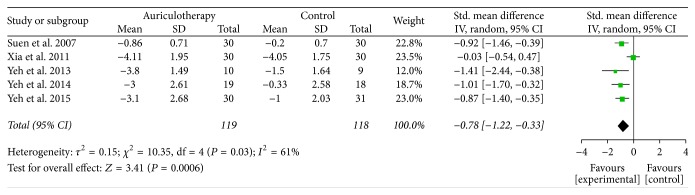
Forest plot of pain relief of AA at 4 weeks.

**Figure 4:**
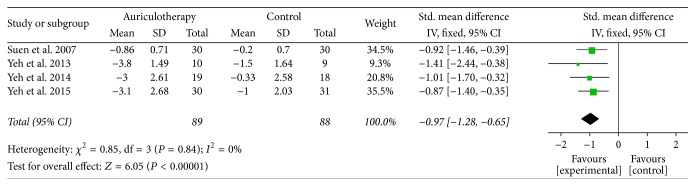
Forest plot of sensitivity analysis (pain relief of AA at 4 weeks).

At 4 weeks and similar cases, there were 5 RCTs which used BPI-sf, VAS, and VRS as pain intensity scales [20, 36–39], and the data of changes from baseline were available or calculated. The result of meta-analysis revealed that, compared with control group, AA group had a large, significant effect in relieving pain at 4 weeks [SMD = −0.78, 95% CI (−1.22, −0.33), *P* < 0.001] (Figure 3). Due to the obvious statistical heterogeneity, a sensitivity analysis was performed. After removing the study of Xia et al., the *I*^2^ value obviously decreased (from 61% to 0%) and the result was relatively stable, where the comparison still statistically favored the AA intervention [SMD = −0.97, 95% CI (−1.28, −0.65), *P* < 0.001] (Figure 4).

**Figure 5:**
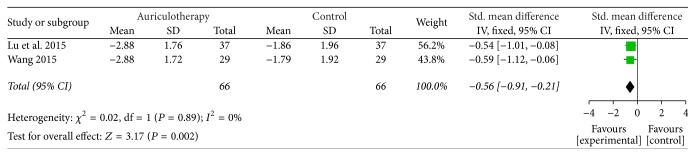
Forest plot of pain relief of AA at 12 weeks.

At 12 weeks, there were 2 RCTs which used VAS and VRS as pain scales [35, 40], and the data of changes from baseline were calculated. The result of meta-analysis revealed that, compared with control group, AA group had a large, significant effect in relieving pain at 12 weeks [SMD = −0.56, 95% CI (−0.91, −0.21), *P* = 0.002] (Figure 5).

**Figure 6:**
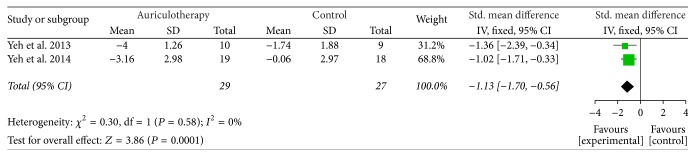
Forest plot of pain relief of AA at 4-week follow-up after 4-week intervention.

*The Follow-Up Effect after the Same Intervention Duration*. During the 4-week follow-up after 4-week intervention, there were 2 RCTs which used BPI-sf and VRS as pain intensity scales [37, 39], and the data of changes from baseline were calculated. The result of meta-analysis revealed that, compared with control group, AA group had a large, significant effect in relieving pain after 4-week follow-up [SMD = −1.13, 95% CI (−1.70, −0.56), *P* < 0.001] (Figure 6).

#### 3.5.2. The Disability Level related to Chronic LBP

Only 3 RCTs assessed the impact of back pain on participants' daily physical functioning [20, 37, 39]. Roland-Morris Disability Questionnaire (RMDQ) disability scale was the most frequently used instrument for disability. Scores of RMDQ ranged from 0 to 24, with higher scores indicating greater disability.

**Figure 7:**
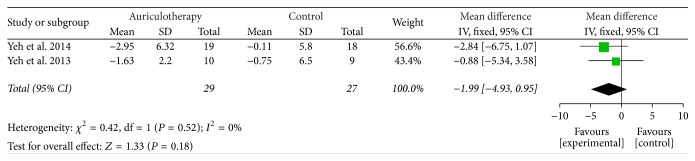
Forest plot of effects of AA on LBP-related disability at 4 weeks.

At 4 weeks, there were 2 RCTs which used RMDQ scale as disability instruments [37, 39], and the data of changes from baseline were calculated. The result of meta-analysis revealed that, compared with control group, AA group had no statistically significant effect in reducing disability but a favorable trend at 4 weeks [MD = −1.99, 95% CI (−4.93, 0.95), *P* = 0.18] (Figure 7).

**Figure 8:**
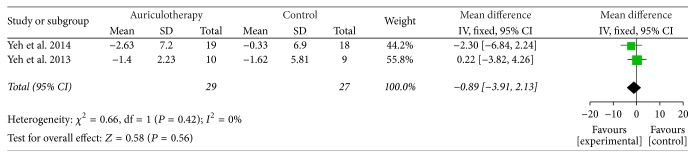
Forest plot of effects of AA on LBP-related disability at 4-week follow-up after 4-week intervention.

At 4-week follow-up after 4-week intervention, there were 2 RCTs which used RMDQ as disability scale [37, 39], and the data of changes from baseline were calculated. The result of meta-analysis revealed that, compared with control group, AA did not reduce disability significantly at the end of 4-week follow-up [MD = −0.89, 95% CI (−3.91, 2.13), *P* = 0.56] (Figure 8).

The remaining 1 study used the modified Aberdeen low back pain scale to assess disability [20], and the result showed that, compared with control group, AA group demonstrated significant improvement in the overall disability level at the time when therapy was completed, 2- and 4-week follow-up periods.

#### 3.5.3. The Effect of AA for Chronic LBP

**Figure 9:**
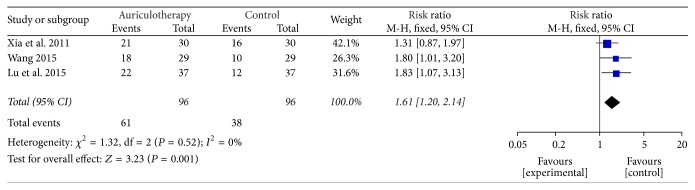
Forest plot of improvement rate of AA for LBP.

*The Improvement Rate of AA for LBP*. According to the guidelines [30, 31], “improvement” criteria referred to the disappearance or obvious relieving of backache and the related stiffness accompanied by freely moving the lower back. Three RCTs used the “improvement” criteria for LBP [35, 36, 40]. And the number of improved cases could be available. The result of meta-analysis revealed that, compared with control group, AA group had a significant effect in managing LBP [RR = 1.61, 95% CI (1.20, 2.14), *P* = 0.001] (Figure 9).

**Figure 10:**
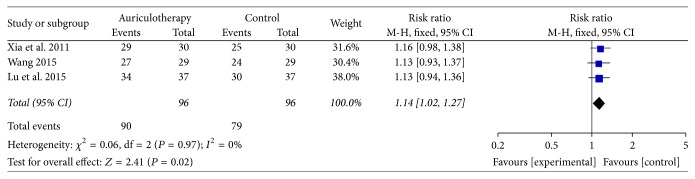
Forest plot of total effective rate of AA for LBP.

*The Total Effective Rate of AA for LBP*. “No effect” was defined as no improvement or even LBP or the stiffness associated with LBP getting worse and the number of total effective could be calculated [30, 31]. Three RCTs used the criteria of “no effect” for LBP [35, 36, 40]. The result of meta-analysis revealed that, compared with control group, AA group had a significant effect in relieving LBP [RR = 1.14, 95% CI (1.02, 1.27), *P* = 0.02] (Figure 10).

### 3.6. Adverse Effects in AA

Five RCTs described the adverse effects of AA during the LBP treatment [35–39]. Xia et al. reported that five patients (5/30) in experimental group felt obvious but tolerable pain of the ears after adopting AA [36]. Three articles expatiated on the uncomfortable symptoms caused by the adhesive tape, such as soreness, sensitization, itch, or sleep disturbance, but all were tolerable [37–39]. And nobody dropped out due to auriculotherapy-related adverse events. Lu et al. reported that there was no adverse effect during auricular therapy [35]. No other adverse events were recorded in the 7 RCTs.

### 3.7. Publication Bias

We did not determine publication bias by funnel plot analyses due to insufficiency of the studies (each comparison included less than 10 trials).

## 4. Discussion and Conclusion

### 4.1. Discussion

On the whole, this study illustrated that AA probably has favorable effects on chronic LBP when compared with other agents. Specifically, for the chronic back pain intensity, results of meta-analyses show that AA has a large immediate effect in reducing pain within 12 weeks and also has a large follow-up effect at 4-week follow-up after 4-week intervention. By contrast, for the disability level related to chronic LBP, results of meta-analyses did not show any statistical significance.

The findings of several recent published meta-analyses of AT for pain management are somewhat consistent with each other with the conclusion that AA has shown promising effects in reducing chronic pain [23, 24, 26]. To our knowledge, data of these previous studies were typically meta-analyzed as quantitative syntheses at final time points, ignoring the different time points across primary studies. It is hard to interpret the pooled value due to the variable final time points, and it is also unfavorable to capture the trend of effectiveness of AA in relieving chronic pain over time [33], while, in our study, all time-points meta-analysis is developed with the aim of combining available evidence at successive time points. For the immediate effect after AA intervention, the pooled estimates from the meta-analysis suggest a large, sustained effect over time (at 4-week and 12-week intervention), while, for the 4-week follow-up effect, compared with the immediate effect at 4-week intervention, the pooled estimates show that AA can produce a positive lasting effect on pain relief. By this approach, the time effectiveness of AA for pain management can be efficiently analyzed, thus giving us a more concrete picture on the role of AA in managing chronic LBP conditions than before.

For the safety of AA in managing chronic LBP, there are seldom reports which revealed the adverse events in the process of adopting AA. In our review, obvious pain of the ears during the LBP treatment and uncomfortable symptoms (i.e., soreness, itch, or sleep disturbance) caused by the adhesive tape were two major complaints, but both were tolerable. Nobody withdrew from the programs due to auriculotherapy-related adverse events. There is insufficient evidence to prove that auricular acupressure is unsafe for patients with chronic LBP.

As is referred above, AA is part of a set of therapeutic techniques based on the principles of TCM which has been widely used as a complementary strategy in the preventive and curative aspects of healthcare. How does AA benefit patients with chronic LBP? Currently, two main lines of research in AA can explain the principles. Firstly, based on TCM, LBP is defined as the obstruction of* Qi* and blood in the meridians which can be caused by external trauma, internal deficiency of antipathogenic* Qi*, or the invasion of exogenous pathogenic factors such as wind-cold or cold-damp [43, 44]. Auricular acupressure, as a noninvasive therapy, focuses on achieving the balance of* yin-yang* and maintaining the function of internal organs through regulation of the* Qi* and blood in the body. Overall, AA can put all of those mentioned above in a state of unity of balance and coordination. Secondly, the French school of AT determined the somatotopic arrangement of external ear as an inverted fetus and theorized that the auricular microsystem may be regarded as reflexology of a neurological action [45–47]. Stimulation of a peripheral reflex point in the auricle is activated along neuron fibers from the auricle to the brain and from the brain through the spinal cord to the correspondent region of the body. This may work due to the fact that groups of pluripotent cells contain information from the whole organism tempting to create regional organization centers representing somatic different parts [48]. These complex nervous interactions may explain the action of AA and are responsible for reducing pain in distal organs. In the last decades, an increasing number of scientific data on the mechanism of AA treatment are corroborated. According to Kumar et al. & Takeshige et al., analgesia induced by needle insertion to auricular acupoints can be blocked by the opiate antagonist naloxone [49, 50], suggesting that a descending pain inhibitory system can be associated with the endorphinergic pathway in the brain and spinal cord. This may help to confirm the role of endorphinergic systems in understanding the underlying mechanisms of auricular therapy. Additionally, in neural pathways modulated by AA, levels of cortisol, serotonin, and norepinephrine also play an important role [51]. Specifically, Santoro and his colleagues claimed that auricular acupressure can increase pain threshold in pain management [52]. This viewpoint verifies the efficacy of auricular acupressure treatments on pain perception. All these factors above may explain the positive role of AA in managing chronic pain.

As a noninvasive and self-managed approach, AA is well accepted by the clients. Compared with other models, the major advantage of auricular acupressure is that patients themselves can stimulate the acupoints by pressing the botanical plant seeds or magnetic pellets taped on the ears with the thumb and forefinger. This implementation emphasizes the importance of patient involvement, collaborative care between patient and healthcare professional, rather than one-way passive care from expert to patient. Results of meta-analysis show that auricular acupressure can provide significant pain relief, which is consistent with the findings of others, thus illustrating that adopting noninvasive model of AA can directly achieve acupuncture-like effects. However, it is worth noting that the implementation approach including selection of acupoints, use of taped objects, instructions of manual pressing, treatment duration, and parents' compliance on AA may partially contribute to the varying therapeutic effect [24]. In terms of acupoints selection, there are still no standardized principles of selecting auricular points yet. Among 93 AAPs developed by the WFAS, one-third were based on the nomenclature and locations of Nogier and 40.8% were based on the Chinese standard which integrated Oleson's zone nomenclature system and emphasized clinical practicability. The remaining acupoints were based on the anatomical terminology of the auricular surface. In this meta-analysis,* Shenmen* (one acupoint based on Chinese model) and subcortex (one acupoint based on Nogier's theory) are the most commonly used acupoints for pain treatment. This coincides with the view of Wang et al., where the nomenclature and acupoints selection should be based on the principle of integration of Chinese and Western Medicine [41], and the positive outcomes for 57.1% (4/7) adopting a sham comparison in our study may suggest that auricular acupoints are indeed specific to particular diseases or symptoms. Nowadays, studies have established evidence of auricular acupressure, yet more RCTs on standardized monitoring parameters of these influential aspects are warranted to establish an adequate assessment of implementation.

As mentioned above, the majority (6/7; 85.7%) of selected RCTs were published in recent years, from 2011 to 2015 [35–40], thus indicating that standardized research on effectiveness of AA in managing chronic LBP has just started. It is not easy to search for powerful original evidence. Only 2 RCTs were rated highly in key domains being considered at low risk of overall bias. The other 5 eligible RCTs were not methodologically rigorous in terms of random allocation, allocation concealment, application of blinding, and record of incomplete outcome data [20, 35–37, 40]. Additionally, with regard to the sample size, in our study, no RCTs were considered to be at low risk of bias (≥200 participants), five RCTs (71.4%) were at an unknown risk of bias (50–200 participants), and 2 RCTs (28.6%) were at a high risk of bias (<50 participants) [53]. These methodological weaknesses may lead to an overestimation of treatment effects. Under such circumstances, in order to improve the power of conclusions, some special individualized strategies, like narrowing down the inclusion criteria and performing critical appraisal of quality, were adopted in this study. Certainly, during the design phase of future RCTs on this topic, those identified methodological flaws will have to be taken into consideration.

There are some limitations of this review. The first concerns the limited number of studies for analysis, especially for ATM. Only seven eligible RCTs were evaluated and there were only two or three RCTs included in some meta-analyses; thus interpreting and generalizing the findings should be cautious. Secondly, the original evidence is not powerful on the whole considering the small sample sizes. And, to our knowledge, some study parameters of implementation (i.e., selection of acupoints, instructions of manual pressing, and duration of AA) are confirmed to be crucial influential factors for therapeutic effect which can impact the overall quality of the RCTs. In the future, we hope systematic review can be updated based on more rigorous and powerful evidence. Thirdly, the use of different interventions (e.g.,* Tai Chi* exercise, walking training, and placebo) in controls may prevent us from drawing firm conclusions about the effectiveness of AA. Moreover, only published studies are included in this study. Leaving the unpublished negative results out of consideration may lead to the less powerful results.

### 4.2. Conclusion

In summary, it is evidenced that, as a relatively safe strategy for pain management, auricular acupressure benefits chronic LBP condition. AA has a large effect in reducing pain within 12 weeks and at 4-week follow-up. For the disability level, the therapeutic effect is not significant when compared with other agents. Overall, there is a pressing need for further rigorously designed large-scale RCTs on chronic LBP for improving pain and disability.

## Figures and Tables

**Table 1:** Methodological quality of all included studies.

Studies	Random allocation	Allocation concealment	Blinding	Incomplete outcome	Selective reporting	Other bias	Quality level
Lu et al. 2015	+	?	−	−	−	+	B
Suen et al. 2007	?	?	+	+	+	+	A
Wang 2015	+	−	−	−	+	+	B
Xia et al. 2011	?	−	−	−	+	+	B
Yeh et al. 2013	+	+	+	+	−	+	A
Yeh et al. 2014	+	+	+	+	+	+	A
Yeh et al. 2015	+	+	+	+	+	+	A

“+” = criteria “met”; “−” = criteria “unmet”; “?” = criteria “unclear.”

**Table 2:** Data extraction of randomized controlled trials on AA for chronic LBP.

Studies	Participants	Diagnostic criteria for chronic LBP/syndrome differentiation	Control intervention
Lu et al. 2015, China/Henan	Middle-aged and elderly patients with the chronic lumbar muscle strain (*n* = 74), E: 37 (27 M, 10 F), MA: 49.39 ± 5.90 yr, C: 37 (26 M, 11 F), MA: 48.77 ± 6.36 yr	Yes/NM	Tai Chi exercise, respectively, in morning and at night, about 45 minutes per time
Suen et al. 2007, China/Hong Kong	The elderly suffering from LBP (*n* = 60), E: 30 (2 M, 28 F), MA: 82.13 ± 6.87 yr, C: 30 (30 F), MA: 81.23 ± 6.21 yr	Yes/NM	AA with the seeds of *Semen vaccariae*
Wang 2015, China/Henan	Middle-aged and elderly patients with the chronic lumbar muscle strain (*n* = 58), E: 29 (22 M, 7 F), MA: 47.4 ± 6.0 yr, C: 29 (21 M, 8 F), MA: 46.6 ± 6.4 yr	Yes/NM	The walking training of lunge twist, respectively, in morning and at night, about 15 minutes per time
Xia et al. 2011, China/Shenzhen	Outpatients with lumbar strain (*n* = 60), E: 30 (13 M, 17 F), MA: 29.25 ± 10.36 yr, C: 30 (11 M, 19 F), MA: 28.52 ± 10.28 yr	Yes/NM	Appling Gu Tong Tie Gao (1-2 plasters/day, 2 weeks in total)
Yeh et al. 2013, USA	Patients with chronic low back pain (*n* = 19), E: 10 (2 M, 8 F), MA: 45.4 ± 21.8 yr, C: 9 (2 M, 7 F), MA: 49.8 ± 14.4 yr	Yes/NM	Treated with auricular acupressure where ear points were not correlated with CLBP (mouth, stomach, duodenum, and kidney)
Yeh et al. 2014, USA	Older patients with chronic low back pain (*n* = 37), E: 19 (4 M, 15 F), MA: 70.6 ± 4.67 yr, C: 18 (7 M, 11 F), MA: 76.7 ± 7.00 yr	Yes/NM	Treated with auricular acupressure where ear points were not correlated with CLBP (mouth, stomach, duodenum, and eye)
Yeh et al. 2015, USA	Patients with chronic low back pain (*n* = 61), E: 30 (10 M, 20 F), MA: 60.97 ± 17.44 yr, C: 31 (10 M, 21 F), MA: 65.61 ± 16.04 yr	Yes/NM	Treated with auricular acupressure where ear points were not correlated with CLBP (mouth, stomach, duodenum, internal ear, and tonsil)

AA, auricular acupressure; LBP, low back pain; E, experimental group; C, control group; M, male; F, female; MA, mean age; yr, year; NM, not mentioned.

**Table 3:** Implementation of randomized controlled trials on AA for chronic LBP.

Studies	Taped objects	Selected ear points (number) Main ear points (M) and adjunct ear points (A)	Acupoint detection	Manual pressing	Using ears alternately	Duration/follow-up
Lu et al. 2015	SV	M(7): liver, kidney, waist, lumbar, sacrum, cortex, and *Shenmen*A(5): adrenal gland, popliteal space, liver, lung, and spleen	Probe	20 min/day for a total of 20 cycles/time per acupoint until *de qi*	√	12 weeks (3-4 days/session)/NM
Suen et al. 2007	E: magnetic pellet C: SV	M(7): *Shenmen*, kidney, urinary bladder, lumbosacral vertebrae, buttock, liver, and spleen	Electronic	No manual pressure exerted	√	3 weeks (3 days/session)/at two and four weeks
Wang 2015	SV	M(7): liver, kidney, waist, lumbar, sacrum, cortex, and *Shenmen*A(5): adrenal gland, popliteal space, liver, lung, and spleen	Probe	20 min/day for a total of 20 cycles/time per acupoint until *de qi*	√	12 weeks (3-4 days/session)/NM
Xia et al. 2011	SV	M(6): *Ashi point*, lumbosacral vertebrae, kidney, liver, *Shenmen*, and subcortex	NM	3 times/day for a total of 3–5 min/time until *de qi*	√	2 weeks (3-4 days/session)/NM
Yeh et al. 2013	SV	M(4): *Shenmen*, sympathetic, nervous subcortex, lower back	Electronic	≥3 times/day for 3 min/time and 3 minutes whenever experiencing pain	No	4 weeks (5 days/session)/1 month
Yeh et al. 2014	SV	M(7): *Shenmen*, sympathetic, nervous subcortex, popliteal fossa, groove of spinal posterior, sciatic nerve, lumbosacral	Electronic	≥3 times/day for 3 min/time and 3 minutes whenever experiencing pain	No	4 weeks (5 days/session)/1 month
Yeh et al. 2015	SV	M(4): *Shenmen*, sympathetic, nervous subcortex, two zones for lower back which located on the front and back of the ear	Electronic	≥3 times/day for 3 min/time and 3 minutes whenever experiencing pain	No	4 weeks (5 days/session)/NM

AA, auricular acupressure; LBP, low back pain; SV, *Semen vaccariae*; *De qi* refers to patient's subjective feelings of soreness, numbness, distention, heaviness, or hotness; NM, not mentioned.

**Table 4:** Therapeutic outcomes of randomized controlled trials on AA for chronic LBP.

Studies	Main outcome measure	Main results	Adverse events
Lu et al. 2015	(1) Pain intensity (VAS) (2) The total effective rate	(1) Significant decrease, *P* < 0.05; E: pre-AA 5.37 ± 1.85, post-AA 2.49 ± 1.66; C: pre-Rx 5.42 ± 2.13, post-Rx 3.56 ± 1.74 (2) Significant difference, *P* < 0.05; E: 91.89% versus C: 81.08%	None
Suen et al. 2007	(1) Pain intensity (VRS)	(1) Significant improvement, *P* < 0.001; E: pre-AA 2.73 ± 0.74, post-AA 1.87 ± 0.68; C: pre-Rx 2.47 ± 0.78, post-Rx 2.27 ± 0.58; significant difference between baseline and the other time points (therapy completed, 2-week follow-up, 4-week follow-up) in E group, *P* < 0.001, E: 2-week follow-up, 2.07 ± 0.69, versus 4-week follow-up, 2.20 ± 0.55	NM
Wang 2015	(1) Pain intensity (VAS) (2) The total effective rate	(1) Significant decrease, *P* < 0.05; E: pre-AA 5.24 ± 1.91, post-AA 2.36 ± 1.43; C: pre-Rx 5.31 ± 2.05, post-Rx 3.52 ± 1.76; significant improvement in E and C within groups (*P* < 0.05) (2) Significant difference, *P* < 0.05; E: 93.1% versus C: 82.8%	NM
Xia et al. 2011	(1) Pain intensity (SFMPQ, consisting of PRI, VAS, and PPI)	(1) Significant difference after 3-day and 2-week treatment in PRI, *P* < 0.05; E: pre-AA 13.76 ± 5.49, post-AA (3-day) 10.92 ± 4.95, post-AA (2-week) 6.25 ± 2.17; C: pre-Rx 13.92 ± 5.25, post-Rx (3-day) 12.98 ± 4.87, post-AA (2-week) 7.56 ± 2.31; significant difference after 3-day treatment in VAS, *P* < 0.05; E: pre-AA 5.79 ± 2.06, post-AA (3-day) 4.75 ± 1.69; C: pre-Rx 5.86 ± 1.89, post-Rx (3-day) 5.09 ± 1.51; significant difference after 3-day treatment in PPI, *P* < 0.05; E: pre-AA 3.82 ± 1.24, post-AA (3-day) 2.92 ± 1.17; C: pre-Rx 3.53 ± 1.12, post-Rx (3-day) 3.25 ± 1.20	14 had mild, tolerable, and short-term itchiness (E: 6, C: 8); 5 in E group felt obvious but tolerable pain of the ears after adopting AA
Yeh et al. 2013	(1) Pain intensity (BPI, consisting of worst pain, average pain, and overall pain intensity) (2) Physical functioning (RMDQ)	(1) Significant decrease in “worst pain” between E and C at end of intervention and 1-month follow-up, *P* < 0.01; E: pre-AA 5.40 ± 0.97, post-AA 1.60 ± 1.71, follow-up 1.40 ± 1.43; C: pre-Rx 5.88 ± 1.89, post-Rx 4.38 ± 1.06, follow-up 4.14 ± 1.86; The scores of “average pain” and “overall pain severity” had a similar change in pattern (2) No significant decrease in physical function which was in expected direction, *P* > 0.05; E: pre-AA 3.30 ± 2.54, post-AA 1.67 ± 1.32, follow-up 1.90 ± 1.66; C: pre-Rx 7.75 ± 6.23, post-Rx 7.00 ± 6.74, follow-up 6.13 ± 5.28	Both groups experienced sensitivity (*n* = 3), soreness (*n* = 4), discomfort (*n* = 4), itching on the ear (*n* = 7), and sleep disturbance (*n* = 2), but all were tolerable
Yeh et al. 2014	(1) Pain intensity (2) Physical functioning (RMDQ)	(1) Significant decrease in “worst pain” at end of intervention and 1-month follow-up, *P* < 0.01; E: pre-AA 7.32 ± 2.03, post-AA (−3.00), follow-up (−3.16); C: pre-Rx 7.28 ± 1.90, post-Rx (−0.33), follow-up (−0.06) (2) Significant decrease in physical function, *P* < 0.01; E: pre-AA 11.11 ± 6.11, post-AA (−2.95), follow-up (−2.63); C: pre-Rx 14.11 ± 4.57, post-Rx (−0.11), follow-up (−0.33)	Both groups experienced sensitivity (*n* = 3), soreness (*n* = 4), discomfort (*n* = 4), itching on the ear (*n* = 7), and sleep disturbance (*n* = 2), but all were tolerable
Yeh et al. 2015	(1) Pain intensity (BPI, consisting of worst pain, average pain, and overall pain intensity) (2) Change over time in daily worst pain intensity (3) Relationship of seed pressing time, frequency, and analgesic use to pain intensity change	(1) Significant decrease in “worst pain” between E and C at end of intervention, *P* < 0.01; E: pre-AA 6.90 ± 1.85, post-AA 3.81 ± 3.12; C: pre-Rx 7.13 ± 1.87, post-Rx 6.13 ± 2.93; greatest reduction was recorded in “average pain” in E group: pre-AA 6.87 ± 1.82, post-AA 2.85 ± 2.54 (2) The mean score of worst pain decreased 30.0% after the first day of AA, reached the largest decrease (50.08%) at day 24, and eventually settled at a 47.67% reduction at day 28; three significant changes occurred, respectively, at day 1, day 5, and day 8 (3) Total pressing time, average minutes per pressing, and analgesic use developed a statistically significant association with worst pain intensity (each 1-minute increase in pressing the seeds significantly reduced the worst pain score by 0.04; each 1-minute increase in total amount of time in which the seeds were pressed significantly reduced average pain by 0.05)	11 had soreness and tenderness of the ear and 3 had irritation and sensitization caused by the adhesive tape

AA, auricular acupressure; LBP, low back pain; VAS, Visual Analogue Scale; E, experimental group; C, control group; VRS, Verbal Rating Scale (VRS-Chinese); NM, not mentioned; Rx, treatment; SFMPQ, Short-Form McGill Pain Questionnaire; PRI, pain rating index; VAS, Visual Analogue Scale; PPI, present pain intensity; BPI, Brief Pain Inventory (short form); RMDQ, Roland-Morris Disability Questionnaire.
